# Oxaloacetate Treatment For Mental And Physical Fatigue In Myalgic Encephalomyelitis/Chronic Fatigue Syndrome (ME/CFS) and Long-COVID fatigue patients: a non-randomized controlled clinical trial

**DOI:** 10.1186/s12967-022-03488-3

**Published:** 2022-06-28

**Authors:** Alan Cash, David Lyons Kaufman

**Affiliations:** 1 Terra Biological LLC, 3830 Valley Centre Drive, Ste 705 PMB 561, San Diego, CA USA; 2 Center for Complex Diseases, Seattle, WA USA

**Keywords:** Oxaloacetate, ME/CFS, Post viral fatigue, Chronic fatigue syndrome, ME/CFS treatment, ME/CFS clinical, Anhydrous enol oxaloacetate, Long COVID, COVID fatigue

## Abstract

**Background:**

There is no approved pharmaceutical intervention for Myalgic Encephalomyelitis/ Chronic Fatigue Syndrome (ME/CFS). Fatigue in these patients can last for decades. Long COVID may continue to ME/CFS, and currently, it is estimated that up to 20 million Americans have significant symptoms after COVID, and the most common symptom is fatigue. Anhydrous Enol-Oxaloacetate, (AEO) a nutritional supplement, has been anecdotally reported to relieve physical and mental fatigue and is dimished in ME/CFS patients. Here, we examine the use of higher dosage AEO as a medical food to relieve pathological fatigue.

**Methods:**

ME/CFS and Long-COVID patients were enrolled in an open label dose escalating “Proof of Concept” non-randomized controlled clinical trial with 500 mg AEO capsules. Control was provided by a historical ME/CFS fatigue trial and supporting meta-analysis study, which showed average improvement with oral placebo using the Chalder Scale of 5.9% improvement from baseline. At baseline, 73.7% of the ME/CFS patients were women, average age was 47 and length of ME/CFS from diagnosis was 8.9 years**.** The Long-COVID patients were a random group that responded to social media advertising (Face Book) with symptoms for at least 6 months. ME/CFS patients were given separate doses of 500 mg BID (N = 23), 1,000 mg BID (N = 29) and 1000 mg TID (N = 24) AEO for six weeks. Long COVID patients were given 500 mg AEO BID (N = 22) and 1000 mg AEO (N = 21), again over a six-week period. The main outcome measure was to compare baseline scoring with results at 6 weeks with the Chalder Fatigue Score (Likert Scoring) versus historical placebo. The hypothesis being tested was formulated prior to data collection.

**Results:**

76 ME/CFS patients (73.7% women, median age of 47) showed an average reduction in fatigue at 6 weeks as measured by the “Chalder Fatigue Questionnaire” of 22.5% to 27.9% from baseline (P < 0.005) (Likert scoring). Both physical and mental fatigue were significantly improved over baseline and historical placebo. Fatigue amelioration in ME/CFS patients increased in a dose dependent manner from 21.7% for 500 mg BID to 27.6% for 1000 mg Oxaloacetate BID to 33.3% for 1000 mg TID. Long COVID patients’ fatigue was significantly reduced by up to 46.8% in 6-weeks.

**Conclusions:**

Significant reductions in physical and metal fatigue for ME/CFS and Long-COVID patients were seen after 6 weeks of treatment. As there has been little progress in providing fatigue relief for the millions of ME/CFS and Long COVID patients, anhydrous enol oxaloacetate may bridge this important medical need. Further study of oxaloacetate supplementation for the treatment of ME/CFS and Long COVID is warranted.

*Trial Registration* https://clinicaltrials.gov/ct2/show/NCT04592354 Registered October 19, 2020.

**Graphical Abstract:**

**Figure:**
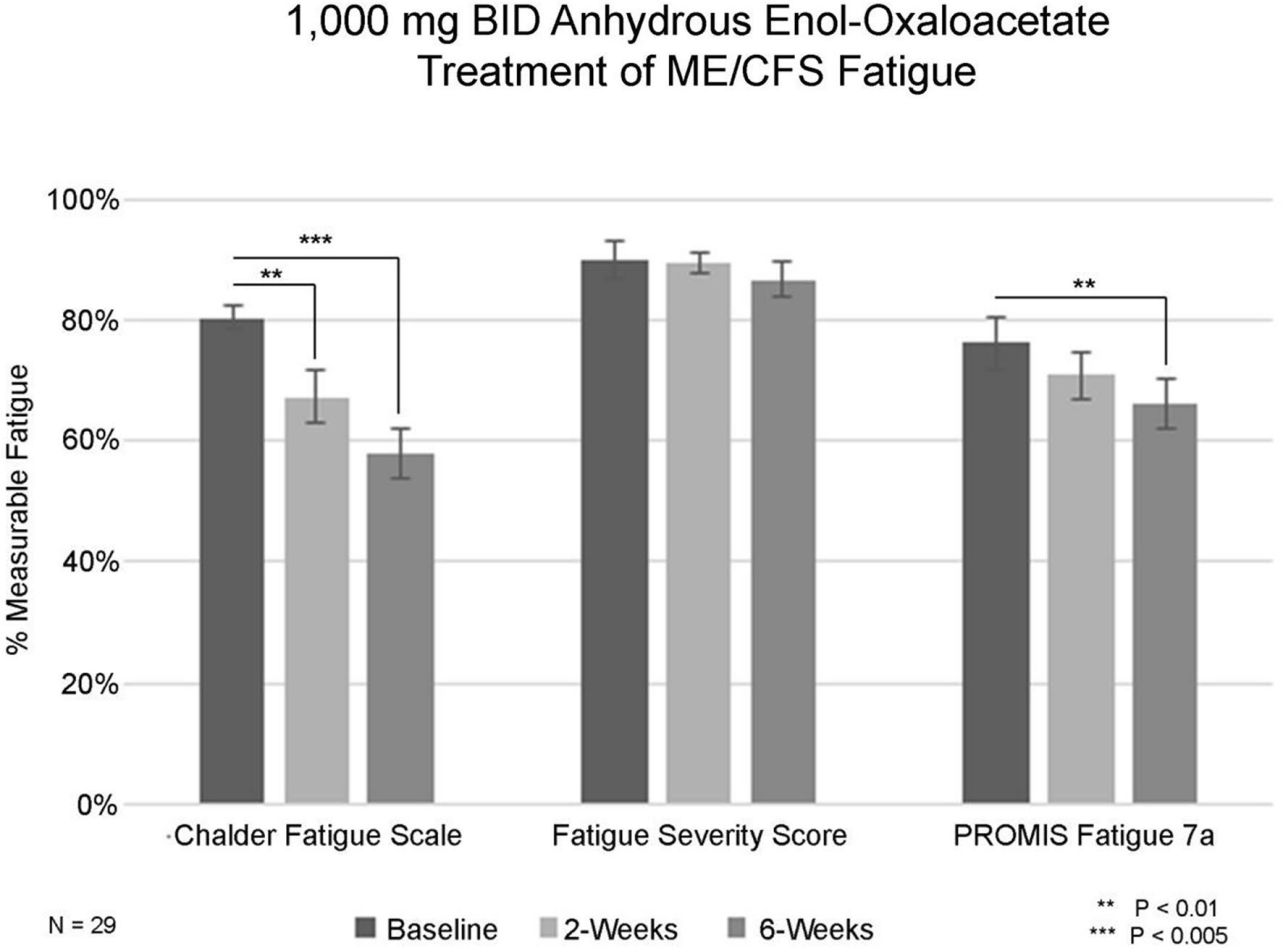
1,000 mg BID Normalized Fatigue Data for Baseline, 2-weeks and 6-weeks evaluated by 3 Validated Fatigue Scoring Questionnaires

## Key points

**Question:** Can normalization of metabolism with oxaloacetate help reduce fatigue in ME/CFS and Long COVID?

**Findings:** Patients with ME/CFS and Long-COVID treated with oral Anhydrous Enol-Oxaloacetate capsules achieved highly significant reductions in physical and mental fatigue within 6 weeks.

**Meaning:** Pathological Fatigue is an unmet medical problem pervasive in ME/CFS, Long-COVID, and other diseases. Here, treatment to normalize metabolism with Anhydrous Enol-Oxaloacetate has for the first time shown improvements in Pathological Fatigue.

## Background

Physiological Fatigue is familiar to most persons, primarily resulting from exertion [1]. It can also be caused by sleep loss or extended wakefulness, disrupted circadian rhythm or increased workload [2]. In contrast, Pathological Fatigue or pathological exhaustion is more than tiredness [3] and refers to physical and mental fatigue that may be caused by viral infection, bacterial infection, trauma, disease, over-work, over-training, epigenetic or genetic alteration that results in physical and mental fatigue that is not improved by bed rest and may be worsened by physical or mental activity.

Physiological Fatigue is caused by neurological changes, calcium level changes, blood flow and oxygen levels, reduced ATP energy levels and glycogen levels, and an increase in intracellular metabolites such as H + , lactate, Pi and ROS  [4]. Most importantly, these physiological changes are reversed by rest.

In contrast, while Pathological Fatigue may involve some of the same physiological changes seen in physiological fatigue, there are many additional metabolic changes that occur in Pathological Fatigue, including changes in energy production pathways, redox of the cells, inflammation response, mitochondrial malfunction, reduced AMPK activation (and related glucose uptake into tissues) and reduced Vitamin D in older subjects [5]. Unlike Physiological Fatigue, the metabolic changes in Pathological Fatigue are not reversed by rest, and the fatigue may, for example, last long after the virus has been conquered, the bacterial invasion has been defeated, or the damaged tissue repaired [6].

Specific examples of this include the disorder Myalgic Encephalomyelitis/Chronic Fatigue Syndrome (ME/CFS) and “Long COVID”. There are many metabolic changes that link ME/CFS and Long COVID [7] and indeed COVID infection may lead to a diagnosis of ME/CFS [8].

Physiological muscle fatigue is easily cured by rest, allowing the nutrients to be taken in by the muscles and waste products such as lactate to be removed by normal cellular processes. In contrast, with pathological fatigue due to damage, whether from viral or bacterial infection, trauma, disease or other cellular assault, the cellular metabolism changes do not always re-set after providing energy for the defense/repair of the body [6]. The failure of metabolism to re-set back to a normal state leads to on-going mental and physical fatigue, which can last for years, even after the original insult to the body is resolved.

### Metabolic changes seen in ME/CFS

Various metabolic mechanisms are turned on by the damage to the body, and these ongoing metabolic changes can cause lasting fatigue if they are not reprogramed back to the original normal metabolic state. Naviaux et al. suggests that these changes are characteristic of the “Dauer” state due to the “cell danger response” [5]. One such metabolic change is the increase in glycolysis in the cytoplasm of the cell. This shift in metabolism was first described by Otto Warburg in the 1930’s and has been named the “Warburg Effect”. Warburg described the metabolic energy shift in relation to cancer cells, and indeed, almost all cancers exhibit this change in energy metabolism. Otto Warburg thought that once the cell changed to this different energy production method, it could not change back into a normal cell. This energy pathway change can lead to pathological fatigue [9].

The Warburg Effect is not only present in cancer cells but is seen in adaptive immune cells of myeloid and lymphoid lineage, characterized by a shift to aerobic glycolysis [10]. The Warburg Effect is present in the replication of viruses such as MERS-CoV and SARS-CoV-2 [11]. Clinical work in ME/CFS patients shows this change to Warburg Effect metabolism, thus generating most of the energy currency, ATP, from non-mitochondrial sources [12].

Another metabolic change seen in fatigued patients is the decrease in the NAD + /NADH in the cytoplasm  [7]. NAD + levels in the cell act as a signaling molecule to drive certain metabolic states. In humans, NAD + levels decrease with muscle use. As an example of this, Graham et. al (1978) found that muscle NAD + levels are decreased with exercise at 65% and 100% of maximal oxygen uptake (V̇o_2 max_), and although increased muscle water accounted for ∼73% of this decrease, NAD + levels were still reduced when assessed on a dry weight basis [13]. NADH levels also increase [14], which further drives down the NAD + /NADH ratio. In contrast with normal patients, Sweetman et. al. (2020) calculated that NADH levels are higher in peripheral blood mononuclear cells in patients with ME/CFS [15].

Yet another metabolic change that takes place in response to cellular stress/damage is the translocation of the protein complex NF-kB from the cytoplasm to the nuclear compartment. While this response is critical for keeping us healthy, in some persons the response does not shut-off, such as in COVID-19 patients with Long-Haul symptoms, and the energy of the cell is continually tied up in immune response [16]. This inflammation pathway change to a chronic state can lead to on-going fatigue and is seen in the diseases that have fatigue as a common determinant [17–19].

Mitochondria are organelles that produce most of the energy during normal cell function. Increased energy demands to fight infection and repair tissues can increase the production of reactive oxygen species (ROS) within the mitochondria, damaging mitochondrial function. Mitochondrial malfunction is implicated in ME/CFS patients [20].

Another metabolic change that takes place in response to cellular stress/damage is reduced activation of the AMPK protein, and a resulting reduction in glucose uptake by tissues. This is seen directly in cells from ME/CFS patients [21]. Reductions in the glucose fuel available to power the cell can be a direct source of fatigue.

Fisicaro et al. identify that ME/CFS patients and Long COVID patients share neuropathophysiological changes that enhance the production of damaging reactive oxygen species, probably from the host response to the initial infection [22] (Table 1).

**Table 1:** Metabolic Changes That May Affect Fatigue in ME/CFS Patients

Metabolic change	Effect on ME/CFS patient	Normalization by oxaloacetate
Warburg Effect	Increased lactate production	Reduction in lactate production via inhibition of lactate dehydrogenase in the cytosol
Decrease in NAD + /NADH ratio	Increase ROS production	Reset of NAD + /NADH ratio and quenching of ROS by antioxidant Oxaloacetate
Increased NF-kB movement to nucleus	Activation of chronic inflammation	Reset of inflammation pathway to normal by Lowering NF-kB translocation to nucleus
Mitochondrial damage	Reduced ability to process glucose	Increased number of mitochondria to produce energy via PGC1-alpha increase
Reduced AMPK activation	Reduced cellular glucose uptake	Increase in glucose uptake via AMPK activation and more glucose fuel available for the patient
Increased neurological ROS production	Damage from free radicals	Oxaloacetate is a highly effective antioxidant

These six changes in metabolism seen in ME/CFS patients are different from what is seen in normal controls with physiological fatigue.

One method of decreasing fatigue may be to address these cellular dysfunctional metabolic changes and move them back towards normal functioning. Oxaloacetate, a human energy metabolite, has been shown to increase muscle endurance and reduce muscle fatigue in normal cells that have fatigue stimulated by muscle overuse via electrical current applied to the muscle [23]. Interestingly, metabolomic studies in ME/CFS patients vs. normal controls indicate that oxaloacetate levels are significantly reduced in the plasma of ME/CFS patients [24]. This study was extended to post-COVID fatigue patients, due to the similarity between ME/CFS and Long COVID.

#### Rationale of this study

This study was performed as there is a medical need for the treatment of pathological fatigue in ME/CFS and Long-COVID.

#### Aim of this study

To see if anhydrous enol-oxaloacetate (AEO) can reduce physical and mental fatigue in ME/CFS and Long-COVID patients.

#### Experimental hypothesis of this study

AEO has been shown to modify many of the metabolic irregularities that are also seen in ME/CFS and Long COVID patients and is deficit in blood serum of ME/CFS patients. Normalization of metabolism with oral AEO may reverse fatigue in this patient group.

## Methods

ME/CFS Patients that met the Fukuda definition [25], agreed to complete a 6-week course of anhydrous enol-oxaloacetate, and to complete three online validated fatigue surveys were selected from the existing patient base of the authors and were offered to join this study. Long-COVID patients that experienced at least 6 months of fatigue and did not have prior fatigue were recruited through social media. Patients completed this trial in their homes.

Patients first completed baseline on-line validated fatigue surveys including the Chalder Fatigue Questionnaire [26], the Fatigue Severity Score [27] and the PROMIS Short Form Fatigue 7A survey [28]. Fatigue surveys were repeated at 2 weeks and 6 weeks for each group. Any adverse events were recorded. Fatigue survey scores were then analyzed for statistical significance. As this was a “Proof of Concept” study, sample groups approximated 23 participants.

After establishing baseline fatigue scores via online fatigue questionnaires, the participants were supplemented with 500 mg anhydrous enol-oxaloacetate (AEO) capsules BID for 6 weeks. After review of any potential side effects indicated safety, the dose was increased in the next patient group to 1000 mg AEO BID for 6 weeks. Finally, after safety review of the 1000 mg AEO BID dose, the dosage of the next group of patients was increased to 1000 mg TID. No incentives were provided.

In this simple study, no stratification of the groups was used. Dose escalation was used with new recruits to the study being placed in the current or next higher dosage group. There was no blinding in this study. The smallest unit that is being analyzed to assess intervention effects is a group of 21 patients.

Fatigue Scores were summarized as means and standard deviations, standard error and confidence intervals were calculated. Changes were summarized as effect sizes, normalized to a 0–100% scale, wherein 100% is the highest amount of fatigue that can be measured with the survey instrument. Significance was calculated from student’s T Test scores in Excel by comparison to baseline. Clinical significance was measured by reductions to four or less in the Chalder fatigue score using bimodal scoring, and by overall significant reductions in fatigue in all tests.

Comparison was made to a historical placebo group, that also used the Chalder Fatigue Score and used an oral placebo. Wearden et. al used an oral capsule in a randomized placebo-controlled trial. 34 patients were given placebo, 5 dropped out of the study. Only two patients (5.9%) of the patients that did not drop out saw clinical improvement on the Chalder Fatigue Questionnaire [29].

While the author Dr. Kaufman had access to many ME/CFS patients due to his specialized practice, and quickly filled the recruitment for the ME/CFS portion of the clinical trial, Long COVID patients were not part of his practice. Participants for the Long COVID trial were recruited via Face Book advertising after meeting recruiting criteria, which included verification of initial COVID infection, verification of COVID infection remission, no historical fatigue prior to COVID infection, and ongoing fatigue for at least 6 months after COVID infection.

All patients in this study provided written acceptance of Informed Consent documents, and that this initial “Proof of Concept” trial was unblinded.

## Results

Participant flow for this trial is shown in the attached diagram. Recruitment began March 2021 and continued into February 2022.
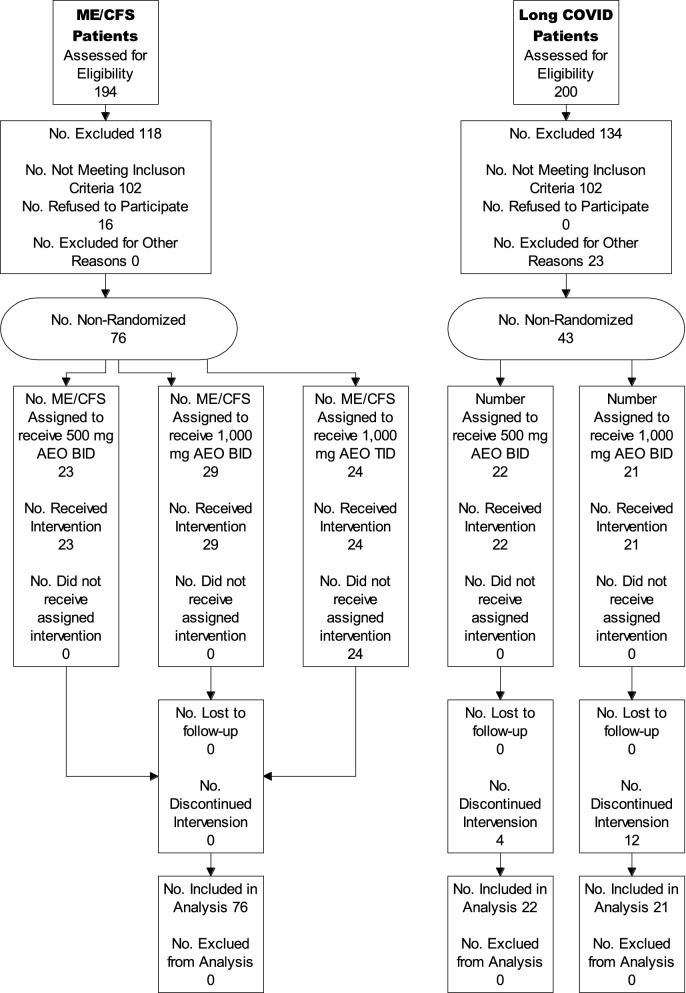


The clinical trial is an open label, dose escalating “Proof of Concept” study for the use of anhydrous enol-oxaloacetate in the treatment of fatigue. It was initially set up with ME/CFS patients then extended to include arms of Long-COVID patients due to the similarities of the two conditions.

Baseline fatigue prior to treatment was assessed by the Chalder Fatigue Scale, the Fatigue Severity Scale and the PROMIS–Fatigue-Short Form 7a. Scores were summarized as mean and standard deviations, standard error and confidence intervals were calculated. Changes are also summarized as effect sizes, normalized to a 0–100% scale, wherein 100% is the highest amount of fatigue that can be measured with the survey instrument. Dose ranging was performed with the first group of patients receiving 500 mg anhydrous enol-oxaloacetate BID, the second group received 1000 mg anhydrous enol-oxaloacetate BID and the third group of ME/CFS patients received 1000 mg anhydrous enol-oxaloacetate TID. The Long COVID patients received 500 mg anhydrous enol-oxaloacetate BID and then 1000 mg anhydrous enol-oxaloacetate BID.

A historical placebo effect in ME/CFS patients was used as a comparator to the data generated in these studies. The historical placebo data showed a Chalder Fatigue Score, Likert scoring, improvement of 5.9% over baseline with an oral intervention placebo over a 26 week period. It is noted that as per a meta-analysis of ME/CFS treatment studies, the placebo effect is small in ME/CFS patients [29].

Initially a total of 76 men and women aged 18–72 with ME/CFS were selected for the clinical trial. An additional 43 patients with Long COVID were added as additional arms of the study. Because women are much more likely to have ME/CFS, most of the ME/CFS patients are women (73.7%). The average length of time that a patient has had ME/CFS is 8.9 years and mean baseline fatigue scores range from 76 to 92% of maximum measurable fatigue for the group (Chalder Fatigue Scale, Likert Scoring). The Chalder Fatigue Scale is often used to measure ME/CFS fatigue. It can be scored in “Likert” method assigning a score range of 0–4 points per question, or it can be scored in “Bimodal” fashion, scoring “0” for normal fatigue levels and “1” for high fatigue levels. After 6 weeks of 500 mg anhydrous enol-oxaloacetate treatment, 5 out 23 patients (21.7%) reduced their measurable fatigue to a score of 4 or less (bimodal scoring), indicating a return from ME/CFS fatigue levels to “normal fatigue” levels. As the average length of ME/CFS illness in this group was over 6 years, seeing > 23% of the participants return to normal fatigue levels with 6 weeks of treatment is very promising. In the 1000 mg AEO BID treatment group, 8 out of 29 patients (27.6%) saw their fatigue drop to 4 or less on the Chalder bimodal fatigue scale. Increasing the dosage to 1000 mg TID, 8 out of 24 patients (33.3%) had fatigue scores drop to 4 or less, showing a consistent dose response of oxaloacetate supplementation vs. drop in clinically relevant fatigue. The reduction in fatigue was also seen with increased time of dosage, improving at 6 weeks over 2 weeks.

Using Likert scoring in the Chalder Fatigue Scale, the Long COVID 500 mg BID group had highly significant reduced fatigue levels from their baseline values by 22.5% in 6 weeks. The Severity Score was highly significantly reduced by 11.7% from baseline, and the PROMIS Fatigue Short Form 7A showed a non-significant 5.9% reduction in fatigue from baseline.

The Long COVID 1000 mg BID group increased effectiveness of the treatment further, with highly significantly reduced fatigue levels by 27.9% from baseline at 6-weesks. The Severity Score showed a non-significant reduction of 2.9%, and the PROMIS Fatigue 7a score was reduced in highly significant fashion by 10.0% within 6-weeks.

In comparison with historical placebo [29], 75.0% of the ME/CFS participants (57/76) saw an improvement in fatigue over what would be expected by placebo effect. 62.8% (27/43) of the Long COVID patients saw improvement over historical placebo.

Not only physical fatigue was improved, but mental fatigue was highly significantly improved in both ME/CFS patients and post-COVID fatigue patients. No ancillary analyses were performed at this time (Tables 2, 3).

**Table 2:** ME/CFS and Long COVID Clinical Relevancy with AEO

ME/CFS and Long COVID Clinical Relevancy with Anhydrous Enol-Oxaloacetate (% of Treated Patients that reduced the Chalder Fatigue Bimodal Score to 4 or less)	
ME/CFS 500 mg BID	21.7%
ME/CFS 1000 mg BID	27.6%
ME/CFS 1000 mg TID	33.3%
Long COVID 500 mg BID	46.8%
Long COVID 1000 mg BID	20.7%

**Table 3:** ME/CFS and Long COVID Improvement in Physical and Mental Fatigue

ME/CFS % Fatigue Improvement From Baseline					
500 mg BID N = 23	Overall Likert Score	Overall Bimodal Score	Treated % at or Below 4 on Bimodal	Physical Fatigue	Mental Fatigue
2-Weeks	18.1%*	25.0%***	21.7%*	16.1%***	19.4%***
6-Weeks	22.5%***	23.7%***	21.7%*	17.3%***	17.5%***

1000 mg BID N = 29	Overall Likert Score	Overall Bimodal Score	Treated % at or Below 4 on Bimodal	Physical Fatigue	Mental Fatigue
2-Weeks	16.3%**	19.7%***	17.1%*	18.7%***	20.5%***
6-Weeks	27.9%***	30.8%***	27.6%***	29.8%***	25.9%***

1000 mg TID N = 24	Overall Likert Score	Overall Bimodal Score	Treated % at or Below 4 on Bimodal	Physical Fatigue	Mental Fatigue
2-Weeks	18.5%**	14.3%*	16.7%*	13.1%*	13.6%*
6-Weeks	23.3%***	26.7%**	33.3%**	24.3%**	18.6%*

	Long COVID Improvement Over Baseline					
	Chalder Fatigue at 6 Weeks					
	2-Weeks	6-Weeks	Physical	Mental	Bimodal	4 or Below
500 mg BID	34.6%***	36%***	33.5%***	23.1%***	46.8%***	45.5%***
1000 mg BID	17.8%*	25.3%***	15%*	2.60%	20.7%*	23.8%**

*P < 0.05

**P < 0.01

***P < 0.005

### Adverse effects

No severe adverse effects were seen in the study. Non-severe adverse effects included Dyspepsia (2/23 in the 500 mg BID group and 2/24 in the 1000 mg BID group) and Insomnia (1/26 in the 500 mg BID group) for ME/CFS patients. In the Long COVID-Fatigue patients, no severe adverse effects were seen. Non-severe adverse effect included stomach upset, headache and constipation in 4/43 patients.

## Discussion

The results document a 21.7–33.3% highly significant improvements in fatigue over baseline with the supplemental addition of 500 and 1000 mg anhydrous enol-oxaloacetate BID and TID in ME/CFS patients within 6-weeks. In post-COVID fatigue patients, improvements of up to 46.8% over baseline were seen (Chalder Fatigue Bimodal Score). Improvement at the higher dosage of 1000 mg BID in Long-COVID patients was lower due to 9 out of 22 dropping out of the trial, and the continuation of the previous data forward. Without the addition of the dropped-out scores, Chalder Fatigue (Likert Scoring) decreased 47.5%. The high dropout rate was probably due to the recruitment of the COVID portion of the trial from social media, no bonus payment for retention, and four capsules had to be taken each day.

We hypothesize that the improvements in fatigue may be due to the normalization of dysfunctional metabolic pathways. Below, we discuss several effects of oxaloacetate on metabolic pathways identified in human and animal studies, and why repletion of oxaloacetate with a medical food may help fatigue amelioration.

### Aberrant energy production via increased glycolysis in the “Warburg Effect”

Cells from persons with ME/CFS show aberrant energy production, wherein more energy is produced within the cytoplasm via increased glycolysis and fermentation [12]. Oxaloacetate has been shown to reverse this trend in human cancer cells, reducing both glycolysis and the formation of lactate [30]. The “Warburg Effect” refers to a form of modified cellular metabolism, which tend to use specialized fermentation of pyruvate to lactate in the cytoplasm over the aerobic respiration pathway that burns pyruvate in the mitochondria that is used by most cells in the body under non-pathological conditions. Chronic Fatigue Syndrome patients have been shown to have activated this alternative energy pathway, increasing the amount of energy that is produced by glycolysis in the cytosol that continues after their pathological incident has passed [12, 31].

### Cells from patients with fatigue show significantly lower NAD + /NADH ratio levels

Oxaloacetate increases the NAD + /NADH ratio in animal models  [32, 33] which would push this ratio in ME/CFS patients towards normalization. When oxaloacetate enters the cell, it can react to the metabolite “malate” in the cytoplasm via the action of the ubiquitous enzyme malate dehydrogenase. As part of this reaction, NADH is turned into NAD + , boosting the NAD + /NADH ratio. Krebs measured the change in the NAD + /NADH ratio with supplemental oxaloacetate as a 900% increase within 2 min [34].

### NF-kB inflammation reduction

Cells from persons with ME/CFS show increased activation of NF-kB leading to persistently elevated levels of inflammatory proteins [19]. This inflammation pathway change can lead to on-going fatigue and is seen in the diseases that have fatigue as a common determinant [17–19]. Oxaloacetate has been shown to reduce the activation of NF-kB by up to 70% in animal models [32]. The reduction in NF-kB overactivation may lead to significant reductions in chronic inflammation and fatigue.

### Mitochondrial damage is prevalent in fatigued patients [35] 

In normal cells, the mitochondria are the “powerplants” of the cells. Oxaloacetate upregulates PGC-1alpha, which in turn activates mitochondrial biogenesis, leading to increased mitochondrial density [36]. Having more powerplants to burn glucose, and replace defective mitochondria, may be a major factor in reducing fatigue.

### AMPK activation reduction

Cells from persons with disabling fatigue show an impaired AMPK activation, and impaired stimulation of glucose uptake [21, 37]. AMPK is an energy sensing protein, which is activated during energy shortages in normal cells. Failure to activate AMPK will result in a reduction in glucose uptake by tissues. The reduced fuel available to the cell can be a direct source of fatigue. Oxaloacetate has been shown to increase glucose uptake in trials with diabetic patients and Alzheimer’s patients [38, 39] providing a mechanism to increase the amount of fuel available for cellular functions.

### ROS reduction

Oxaloacetate is a powerful antioxidant, reducing both thiobarbituric acid and hydrogen peroxide in the brain [40, 41]. Oxaloacetate also protects mitochondrial DNA from damage from agents such as Kainic acid [42].

These six metabolic changes in ME/CFS and other fatigue patients may be the driving force of fatigue. Normalization of these metabolic changes by oxaloacetate may restore a non-fatigue state.

There were several limitations to this “Proof of Concept” study. The study was unblinded, as it initially started out as “case studies” using author Dr. Kaufman’s ME/CFS patient group. Positive results led to more patient participation and formal recognition of this as a clinical trial with IRB oversight. As there was no placebo group (only historical placebo), there was no randomization into a separate group. Face Book advertising identified many potential Long-Haul candidates for this study, but as there was no payment to these patients, and the patients did not have an ongoing relationship with either of the authors of this study, drop-out rates were higher in the Long COVID groups than in the ME/CFS groups. This is a small Proof of Concept study, and even though very high significance was found for fatigue reduction, clinical investigations should be continued.

## Conclusion

This small, non-randomized open-label dose escalating “Proof-of-Concept” clinical trial yielded impressive highly significant improvements in fatigue in both ME/CFS patients and Long-COVID patients. Clinical Efficacy was measured by the decrease in the Chalder Fatigue bimodal score from above 4 to 4 or below. Up to 33% of the patients with ME/CFS and up to 46.8% of Long COVID fatigue patients achieved clinical efficacy against fatigue with oral anhydrous enol-oxaloacetate treatment at 6 weeks. This compares well with historical placebo that achieved 5.9% clinical improvement in ME/CFS patients. Both physical and mental fatigue were significantly improved in both ME/CFS and Long COVID fatigue patients. 1000–3000 mg anhydrous enol-oxaloacetate daily was both safe and tolerable in this population for the duration of the trial. This proof-of-concept study supports the further development of anhydrous enol-oxaloacetate for the treatment of ME/CFS patients and Long COVID fatigue patients with longer randomized placebo-controlled studies. Potential clinical applications with anhydrous enol-oxaloacetate, currently a commercial nutritional supplement, may help reduce fatigue in ME/CFS and Long-COVID patients.

## Data Availability

The datasets used and/or analyzed during the current study are available from the corresponding author on reasonable request.

## Abbreviations

AEO: Anhydrous enol-oxaloacetate
AMPK: AMP-activated protein kinase
BID: Dosing twice per day
COVID: Disease caused by a coronavirus called SARS-CoV-2
ME/CFS: Myalgic Encephalomyelitis/Chronic Fatigue Syndrome
NF-kB: Nuclear factor kappa B
NAD+ and NADH: Nicotinamide adenine dinucleotide
PGC-1alpha: Peroxisome proliferator-activated receptor-gamma coactivator -1 alpha
ROS: Reactive oxygen species
TID: Dosing three times per day
